# The Pathogenic Effects of *Fusobacterium nucleatum* on the Proliferation, Osteogenic Differentiation, and Transcriptome of Osteoblasts

**DOI:** 10.3389/fcell.2020.00807

**Published:** 2020-09-11

**Authors:** Hui Gao, Tianyong Sun, Fanghong Yang, Jiakan Yuan, Mei Yang, Wenyan Kang, Di Tang, Jun Zhang, Qiang Feng

**Affiliations:** ^1^Department of Orthodontics, School and Hospital of Stomatology, Cheeloo College of Medicine, Shandong University and Shandong Key Laboratory of Oral Tissue Regeneration and Shandong Engineering Laboratory for Dental Materials and Oral Tissue Regeneration, Jinan, China; ^2^Department of Human Microbiome, School and Hospital of Stomatology, Cheeloo College of Medicine, Shandong University and Shandong Key Laboratory of Oral Tissue Regeneration and Shandong Engineering Laboratory for Dental Materials and Oral Tissue Regeneration, Jinan, China; ^3^Department of Stomatology, Weifang People’s Hospital, Weifang, China; ^4^Department of Stomatology, Heze Municipal Hospital, Heze, China; ^5^Department of General Dentistry, Qingdao Stomatological Hospital, Qingdao, China; ^6^Department of Periodontology, School of Stomatology, Shandong University, Jinan, China; ^7^State Key Laboratory of Microbial Technology, Shandong University, Qingdao, China

**Keywords:** *Fusobacterium nucleatum*, osteoblasts, cell proliferation, inflammation, cell differentiation, osteogenesis, RNA-seq

## Abstract

As one of the most common oral diseases, periodontitis is closely correlated with tooth loss in middle-aged and elderly people. *Fusobacterium nucleatum* (*F. nucleatum*) contributes to periodontitis, but the evidence in alveolar bone loss is still unclear. In this study, cytological experiments and transcriptome analyses were performed to characterize the biological process abnormalities and the molecular changes of *F. nucleatum*-stimulated osteoblasts. *F. nucleatum* could inhibit cell proliferation, promote cell apoptosis, and elevate pro-inflammatory cytokine production of osteoblasts, and it also inhibited osteoblast differentiation and mineralized nodule formation and decreased the expression of osteogenetic genes and proteins. Whole-transcriptome analyses identified a total of 235 transcripts that were differentially expressed in all six time points, most of which were inflammation-related genes. The genes, *Ccl2*, *Ccl20*, *Csf1*, *Cx3cl1*, *Cxcl1*, *Cxcl3*, *Il6*, *Birc3*, *Map3k8*, *Nos2*, *Nfkb2*, *Tnfrsf1b*, and *Vcam1*, played core roles in a PPI network, and interacted closely with other ones in the infection. In addition, 133 osteogenesis-related differential expression genes (DEGs) were time-serially dynamically changed in a short time-series expression miner (STEM) analysis, which were enriched in multiple cancer-related pathways. The core dynamic DEGs (*Mnda*, *Cyp1b1*, *Comp*, *Phex*, *Mmp3*, *Tnfrsf1b*, *Fbln5*, and *Nfkb2*) had been reported to be closely related to the development and metastasis in tumor and cancer progress. This study is the first to evaluate the long-term interaction of *F. nucleatum* on osteoblasts, which might increase the risk of cell carcinogenesis of normal osteoblasts, and provides new insight into the pathogenesis of bacterial-induced bone destruction.

## Introduction

Pathogenic infection is an important cause of bone loss in diseases such as periodontitis, dental cysts, bacterial arthritis, and osteomyelitis (Bolstad et al., 1996; Almstahl and Wikstrom, 1999; Reis et al., 2016). Pathogens such as *Actinobacillus actinomycetemcomitans* and *Porphyromonas gingivalis* (*P. gingivalis*) can produce a group of molecules that have potential effects on osteoblast cells and participate in bone loss (Nair et al., 1996). *Fusobacterium nucleatum* (*F. nucleatum*) has been frequently reported in gingivitis, periodontitis, pulpal necrosis, and dental pulp infection (caused by chronic apical periodontitis) (Siqueira and Rocas, 2009; Reis et al., 2016; Kook et al., 2017). In periodontitis, the detection rate and quantity of *F. nucleatum* are positively correlated with gingival inflammation and periodontal tissue damage (Yang et al., 2014). *F. nucleatum* can recruit oral pathogens and promote their adhesion to the dental biofilm and invasion into the oral tissue (Bolstad et al., 1996; Kolenbrander et al., 2010; Kolenbrander, 2011). In addition, *F. nucleatum* invades epithelial cells and contributes to the development of colorectal cancer (Feng and Weinberg, 2006; Ji and Choi, 2013; Shang and Liu, 2018).

Bone loss caused by pathogen invasion is an important clinical feature of periodontitis (Glowacki et al., 2013). However, the roles of *F. nucleatum* in bone loss are not sufficiently understood. The combination of *F. nucleatum* with *P. gingivalis* or *Tannerella forsythia* causes more severe alveolar bone loss than each of these pathogens alone, and *F. nucleatum* can induce a more severe inflammatory response than *P. gingivalis* (Polak et al., 2009). *F. nucleatum* and *A. actinomycetemcomitans* exert a protective effect against *P. gingivalis*-induced alveolar bone loss in immunization-provoked and bovine collagen type II-induced arthritis (Meinolf et al., 2018).

Periodontal pathogen-induced bone destruction has been reported extensively *in vitro* (Morton et al., 1977; Su et al., 2010; Verdugo et al., 2015). *P. gingivalis* could significantly inhibit the expression of osteogenic transcription factors and inhibit the differentiation of primary mouse osteoblasts but did not affect osteoblast proliferation (Zhang et al., 2010). *A. actinomycetemcomitans* could reduce cell proliferation, increase cell death, and induce osteogenic differentiation in undifferentiated MG63 cells, and matrix metalloproteinase network stability was found to be sensitive to *P. gingivalis* but not *A. actinomycetemcomitans* (Hoppe et al., 2018).

*Fusobacterium nucleatum* may contribute to alveolar bone loss as it maintains a high oral tissue-invasive ability and affects the biological behavior of many oral cell types (Yang et al., 2017; Geng et al., 2020). In our study, calvarial osteoblasts were sequentially stimulated with *F. nucleatum* for a long period and evaluated for their biological activities (cell proliferation, apoptosis, proinflammatory cytokine production, and osteogenic differentiation). Time-course RNA sequencing (RNA-seq) was used to analyze the long-term pathogenic effects of *F. nucleatum* on gene expression in osteoblasts at multiple time points. We established a long-term *in vitro* coculture model of *F. nucleatum* and osteoblasts and studied the changes in osteoblasts under time-series infection of *F. nucleatum*. We additionally found that cancer-related genes were continuously changed in osteoblasts under long-term stimulation of *F. nucleatum.* Our findings provide a foundation for the future study of the molecular mechanisms of long-term bacteria–cell interactions and for the understanding of the pathogenicity of *F. nucleatum*.

## Materials and Methods

### Preparation of Bacteria

*Fusobacterium nucleatum* (ATCC 25586) was provided by the Shandong Key Laboratory of Oral Tissue Regeneration (Jinan, China) and cultured anaerobically in a 37°C incubator (with 80% N_2_, 10% H_2_, and 10% CO_2_) in brain–heart infusion medium (BHI, Haibo, Qingdao, China) containing hemin (5 μg/mL) and menadione (1 μg/mL) for 24–48 h. The cultured bacteria were identified as *F. nucleatum* (ATCC 25586) by polymerase chain reaction (PCR), sequencing of the 16S rRNA gene, and alignment of the 16S rRNA gene sequence in NCBI. The required bacteria were centrifuged at 4000 rpm for 5 min and washed twice with phosphate-buffered saline before being added to osteoblasts. The bacterial density was determined with a spectrophotometer (at 600 nm), and bacteria were diluted to 10^8^ cells/ml.

### Cell Isolation and Culture

Primary osteoblasts from 24 to 48-h-old fetal Wistar rat calvariae were obtained by sequential enzymatic digestion (Utting et al., 2006; He et al., 2009). Newborn Wistar rats were provided by the Shandong University Laboratory Animal Center. All experimental procedures were approved by the Animal Ethics Committee of Shandong University (Jinan, China), and all animal experiments were carried out in accordance with the National Institutes of Health Guide for the Care and Use of Laboratory Animals (NIH publication No. 8023, revised 1978). Calvariae freed from all soft tissues were digested in 5 ml of α-minimum essential medium (α-MEM, BioInd, Israel) containing 0.1% collagenase II (Sigma-Aldrich, St. Louis, MO, United States) and 0.25% trypsin (Invitrogen, Carlsbad, CA, United States) for 20 min at 37°C, and the supernatants were discarded. Then, the precipitates were digested three times for 30 min in 5 ml of 0.1% collagenase II, and digested cells were collected. Cells were cultured in α-MEM containing 20% fetal bovine serum (BioInd) in a 37°C incubator with 5% CO_2_. When the cell monolayer reached 85–90% confluence, cells were trypsinized and passaged to expand the culture in α-MEM containing 10% fetal bovine serum and no antibiotics. Cells at passages 3–4 were used for the following experiments. Immunocytochemical staining (ZSGB-BIO, China) of type I collagen (Col-1) was carried out on day 3, cytochemical detection of alkaline phosphatase (ALP) was carried out on day 7 using a BCIP/NBT alkaline phosphatase color development kit (Beyotime, China), and von Kossa staining (Solarbio, China) for minerals was performed on day 28. All the results suggested that the cultured primary cells had osteogenic differentiation ability.

### Cell Proliferation Assay

Osteoblasts were seeded into 6-well plates (2 × 10^5^ cells/well) in osteogenic inductive medium [α-MEM supplemented with 10% fetal bovine serum, 50 mg/L ascorbic acid, and 10 mmol/L β-glycerophosphate (Sigma)]. *F. nucleatum* suspensions were added to the cell monolayers at optimal multiplicities of infection (MOIs) of 10, 50, 100, and 200 (*F. nucleatum*:osteoblasts = 10:1, 50:1, 100:1, and 200:1) at 37°C in a 5% CO_2_ atmosphere, and control cells were cultured under the same conditions without *F. nucleatum* infection (MOI = 0) (Liu et al., 2015). The cell culture medium containing newly cultured *F. nucleatum* was changed every other day from day 0 to day 8. The cells were washed with phosphate-buffered saline, harvested by trypsinization, and then counted with an automated cell counter (Countstar, Shanghai, China) at 1, 3, 5, 7, and 9 days. To assess the cell proliferation rate, cells were inoculated in 24-well plates (5 × 10^4^ cells/well) and treated with *F. nucleatum* for 24 h. A 5-ethynyl-20-deoxyuridine (EdU) labeling assay was used to evaluate the cell proliferation ratio according to the instructions of the EdU Apollo DNA *in vitro* kit (RiboBio, Guangzhou, China).

### Cell Apoptosis Analysis

Osteoblasts were seeded into 6-well plates (2 × 10^5^ cells/well) and stimulated with *F. nucleatum* (MOIs = 0, 10, 50, 100, and 200). Cell apoptosis was analyzed at separate time points (2, 6, 12, and 24 h) according to the instructions of the Annexin V-FITC/PI Kit (Dojindo, Kumamoto, Japan). Cells were trypsinized, washed with phosphate-buffered saline, stained with a propidium iodide-conjugated anti-Annexin V antibody for 15 min in the dark, and then subjected to flow cytometry with a FACScan flow cytometer (Becton Dickinson, Franklin Lakes, NJ, United States).

### Secreted Inflammatory Cytokine Measurement by Enzyme-Linked Immunosorbent Assay (ELISA)

Osteoblasts were seeded into 6-well plates (2 × 10^5^ cells/well) and stimulated with *F. nucleatum* (MOIs = 0, 10, 50, 100, and 200) for 24 h. The cell culture supernatants were collected and centrifuged at 12,000 rpm for 5 min at 4°C for subsequent experiments. The levels of secreted interleukin (IL)-6 and tumor necrosis factor alpha (TNF-α) in the supernatants were measured by an enzyme-linked immunosorbent assay kit (Dakewe Biotech, Shenzhen, China).

### Osteogenesis Differentiation Assay

Osteoblasts (5 × 10^4^ cells/well) were seeded into 6-well plates and cultured in osteogenic inductive medium for 28 days. *F. nucleatum* suspensions at MOIs of 0, 10, 50, 100, and 200 were used to stimulate cells every 2∼3 days for 3, 7, 14, 21, and 28 days.

### ALP Activity and Calcium Content Assays

On days 3, 7, 14, and 21, cells were lysed with RIPA buffer for 30 min on ice, and the protein concentration was measured according to the instructions of a bicinchoninic acid protein assay kit (CWBIO, Jiangsu, China). The activity of ALP (at days 3, 7, and 14), an early osteogenic marker (Birmingham et al., 2012), was detected following the instructions of an ALP activity assay kit (Nanjing Jiancheng Bioengineering Institute, Nanjing, China), and the absorbance at a wavelength of 520 nm was measured with a microplate reader (SPECTROstar Nano, Germany). The calcium content (at day 21) in the extract was used as a late osteogenic marker (Salim et al., 2004) and measured colorimetrically using a calcium quantification kit (Nanjing Jiancheng Bioengineering Institute). Then, the absorbance at a wavelength of 620 nm was measured with a microplate reader.

### Alizarin Red Staining

On days 21 and 28, cells were fixed in 4% paraformaldehyde for 10 min and washed three times with phosphate-buffered saline. Then, extracellular matrix calcification nodules were stained with 2% (wt/vol) alizarin red (pH 4.3, Sigma-Aldrich). After rinsing with ddH_2_O, the mineralized nodules were scanned in six-well plates and observed under a light microscope (Olympus, Tokyo, Japan). ImageJ 1.43 software (NIH, Bethesda, MD, United States) was used to analyze the positive area of alizarin red staining (Gee et al., 2020).

### Quantitative Real-Time PCR (qRT-PCR)

Total RNA was extracted from cells after the different treatments with TRIzol (CWBIO, Beijing, China), and 1 μg of mRNA from each sample was reverse transcribed according to the manufacturer’s recommendations to obtain cDNA. Then, qRT-PCR was performed using UltraSYBR Mixture (CWBIO) and a LightCycler 96 PCR system (Roche, Basel, Switzerland). The mRNA expression levels of caspase-8, alkaline phosphatase (biomineralization associated) *(Alpl)*, *Col-1*, runt-related transcription factor 2 (*Runx2*), osteoprotegerin (*Opg*), osterix (*Sp7*/*Osx*), bone sialoprotein (*Bsp*), osteocalcin (*Ocn*), receptor activator of nuclear factor-κB ligand (*Rankl*), cytochrome p450 family 1b1 (*Cyp1b1*), myeloid nuclear differentiation antigen (*Mnda*), cartilage oligomeric matrix protein (*Comp*), phosphate-regulating gene with homologies to endopeptidase on the X chromosome (*Phex*), matrix metalloproteinase 3 (*Mmp3*), tumor necrosis factor-receptor superfamily member 1b (*Tnfrsf1b*), fibulin-5 (*Fbln5*), nuclear factor kappa B subunit 2 (*Nfkb2*), and β-actin (primer sequences are listed in Supplementary Table S1) were detected. The 2^(–ΔΔCT)^ method was used to analyze gene expression levels, and β-actin was used as a housekeeping gene to normalize the data.

### Western Blot Analysis

Cells were washed with phosphate-buffered saline and lysed with RIPA buffer containing 1% PMSF (Solarbio) and 1% phosphatase inhibitor (Solarbio) for 30 min on ice. After measurement of the protein concentration, the proteins (20 μg/lane) were separated by electrophoresis on 10% SDS-polyacrylamide gels and electrotransferred to polyvinylidene fluoride membranes (Millipore, Billerica, MA, United States). Next, the membranes were blocked with 5% non-fat milk for 1 h and incubated with primary antibodies (Supplementary Table S2) at 4°C overnight. Then, horseradish peroxidase-labeled secondary antibodies were added to the membranes (1:5000; Proteintech, Chicago, IN, United States) for 1 h. After a washing step, the western blot signals were imaged by an extrasensitive system (Amersham Imager 600; GE Healthcare Life Sciences, Pittsburgh, PA, United States) with enhanced luminescence reagent (Millipore). ImageJ 1.43 software was used to quantify the protein expression levels.

### Statistical Analysis

All of the cytology experiments were repeated independently three times with cells from three different donors, and the data are expressed as the mean ± standard deviation (SD). GraphPad Prism 6.0 software (MacKiev, Boston, MA, United States) was used for analysis. Data were analyzed by one-way ANOVA or two-way ANOVA followed by Tukey’s honestly significant difference comparison test. The variation between two groups at different times was compared by multiple *t*-test. A *p*-value < 0.05 indicated statistical significance.

### RNA Sequencing

To evaluate changes in osteoblasts under *F. nucleatum* stimulation at the gene expression level, 36 samples from three individuals without (MOI = 0) or with *F. nucleatum* infection (MOI = 50) cultured in osteogenic inductive medium for 1, 3, 7, 14, 21, and 28 days were analyzed by RNA-seq at LC-BIO (LC-BIO, Hangzhou, China). Total RNA was extracted and analyzed with a Bioanalyzer 2100 and RNA 1000 Nano LabChip Kit (Agilent, Santa Clara, CA, United States) with RIN number > 7.0. Poly(A) RNA was purified from total RNA (5 μg) using poly-T oligo-attached magnetic beads in two rounds of purification. Then, the mRNA was fragmented and reverse transcribed to create the final cDNA library in accordance with the protocol for an mRNA-seq sample preparation kit (Illumina, San Diego, United States). The average insert size for the paired-end libraries was 300 bp (± 50 bp). Paired-end sequencing was performed on an Illumina HiSeq 4000 platform. Prior to assembly, the low-quality reads (1, reads containing sequencing adaptors; 2, reads containing sequencing primers; 3, nucleotides with a q quality score lower than 20) were removed. After that, a total of 314G bp of cleaned, paired-end reads were produced. Data were submitted to the NCBI GEO database with accession number GSE133223.

### Transcript Quantification and Differential Expression Analysis

HISAT software was used to align reads to the UCSC^1^ Rattus norvegicus reference genome (Kim et al., 2015). The mapped reads were then assembled using StringTie (Pertea et al., 2015). After assembling, all transcriptomes were merged to reconstruct a comprehensive transcriptome using Perl scripts. A comprehensive profile comprising 24096 genes with 36 slides (24096^∗^36 array) was achieved. The edgeR package (Robinson et al., 2010) was used to investigate the differential expression genes (DEGs) between control groups (MOI = 0) and *F. nucleatum*-infected groups (MOI = 50) at each time point, and DEGs were selected with | log2 (fold change)| > 1 and with statistical significance (*q*-value < 0.05). Principal component analysis (PCA) was performed to compare the differences among control groups and all *F. nucleatum*-infected groups. The overlapping genes among the DEGs of compared groups were visualized by the jvenn viewer (Bardou et al., 2014).

### Protein–Protein Interaction (PPI) Analysis and Enrichment Analysis

A protein–protein interaction analysis was described by uploading the DEGs to STRING (von Mering et al., 2005), and genes with no matched gene symbols in the database were deleted. The parameters of the STRING database were defaulted with combined score > 0.4 (medium confidence). The network was visualized by Cytoscape software (version 3.7.2) (Kohl et al., 2011). Cytoscape’s MCODE plug-in (Bandettini et al., 2012) was used to perform key subnetwork analysis on the PPI network, and the parameters were set by MCODE score > 5, degree cutoff = 2, node score cutoff = 0.2, max depth = 100 and k-score = 2. Meanwhile, enrichment analysis of DEGs was applied with STRING. After that, the Metascape database (Zhou et al., 2019) was used to perform enrichment analysis of DEGs either.

### Short Time-Series Expression Miner (STEM) Analysis

Short time-series expression miner analysis is mainly used for clustering, comparing, and visualizing short time series gene expression data (less than or equal to 8 time points) (Ernst et al., 2005). STEM can identify significant temporal expression profiles, accurately and intuitively screen out genes related to these expression profiles, and compare the expression trends of these genes under different conditions. It can extract and highlight mainstream gene expression trends and rank important and mainstream genes in order of significance level of expression trends. STEM software (version 1.3.11) (Ernst and Bar-Joseph, 2006) was used to identify the dynamic gene expression clusters in osteoblasts infected by *F. nucleatum*.

## Results

### *F. nucleatum* Inhibited Cell Proliferation and Promoted Cell Apoptosis and Inflammatory Cytokine Secretion

**FIGURE 1:**
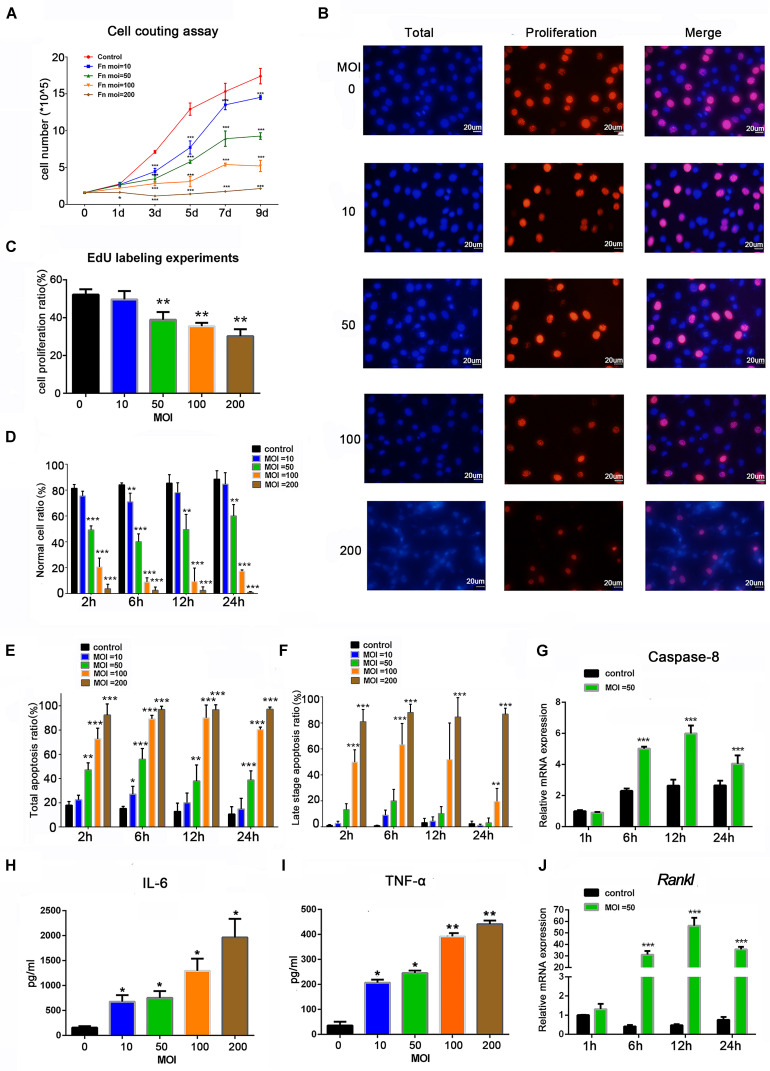
Effects of *Fusobacterium nucleatum* stimulation on cell proliferation, apoptosis, and secretion of inflammatory cytokines in osteoblasts. **(A)** Cell-counting assay of osteoblasts cocultured with *F. nucleatum* at MOIs of 0, 10, 50, 100, and 200 for 1–9 days. **(B)** EdU detection images captured under the microscope. Scale bar: 20 μm. The nuclei labeled by DAPI were blue, and the EdU-labeled proliferating cell nuclei were red and shown pink after merging with the blue marked by DAPI. **(C)** Statistical results of the cell proliferation ratio of osteoblasts detected by EdU assay. Statistical analysis of **(D)** normal cell viability, **(E)** total apoptosis ratio, and **(F)** late-stage cell apoptosis ratio of osteoblasts analyzed by flow cytometry with *F. nucleatum* stimulation (MOIs of 0, 10, 50, 100, and 200) at 2, 6, 12, and 24 h. **(G)** Caspase-8 and **(J)** *Rankl* mRNA relative expression of osteoblasts after *F. nucleatum* stimulation at MOIs of 0 and 50 for 1, 6, 12, and 24 h (control at 1 h = 1). ELISA results of the inflammatory cytokines **(H)** IL-6 and **(I)** TNF-α expression in the cell culture supernatant fluid with *F. nucleatum* stimulation (MOI = 0, 10, 50, 100, and 200) for 24 h. The histograms represent the mean ± SD (*n* = 3) (*p < 0.05; **p < 0.01; ***p < 0.001).

To determine the effects of *F. nucleatum* on cell proliferation, osteoblasts were stimulated with *F. nucleatum* at MOIs of 0, 10, 50, 100, and 200 for 9 days. A cell-counting assay showed that *F. nucleatum* significantly inhibited the rate of osteoblast proliferation in a dose- and time-dependent manner, and the numbers of cells in all *F. nucleatum-*infected groups were significantly decreased from day 3 to day 9 in a dose-dependent manner (Figure 1A). At MOIs of 100 and 200, the proliferation of osteoblasts was almost blocked. Furthermore, an EdU assay at 24 h indicated that both the cell number and cell proliferation rate were dose-dependently reduced by *F. nucleatum* at MOIs of 50, 100, and 200, but the difference in cell proliferation following stimulation with *F. nucleatum* at a low concentration was not significant (Figures 1B,C). The apoptosis ratios of the osteoblasts after 2, 6, 12, and 24 h of *F. nucleatu*m stimulation were detected by flow cytometry (Supplementary Figure S1); *F. nucleatum* at an MOI of 10 had little significant influence on cell apoptosis, while stimulation with *F. nucleatum* at MOIs of 50, 100, and 200 significantly decreased the normal cell ratio and increased the apoptotic cell ratio from 2 to 24 h (Figures 1D,E). The groups infected with *F. nucleatum* at MOIs of 100 and 200 showed a significant reduction in the number of normal cells and an obvious increase in the number of late apoptotic cells (Figures 1D,F). These results indicated that *F. nucleatum* from the MOI of 50 significantly induced the cell apoptosis of osteoblasts. The gene expression of caspase-8, which is closely related to cell apoptosis, was not significantly changed at 1 h but obviously increased from 6 to 24 h of *F. nucleatum* stimulation at MOI of 50 (Figure 1G).

To analyze the effect of *F. nucleatum* stimulation on inflammatory cytokines, the IL-6 and TNF-α protein levels in the cell culture supernatant after 24 h of *F. nucleatum* stimulation were tested (Figures 1H,I), which showed a dose-dependent increase in all groups infected with *F. nucleatum* at an MOI ≥ 10. The gene expression of *Rankl* was low at 1 h, increased at 6 h, peaked at 12 h, and slightly decreased at 24 h (Figure 1J).

### *F. nucleatum* Inhibited the Differentiation and Mineralization of Osteoblasts

**FIGURE 2:**
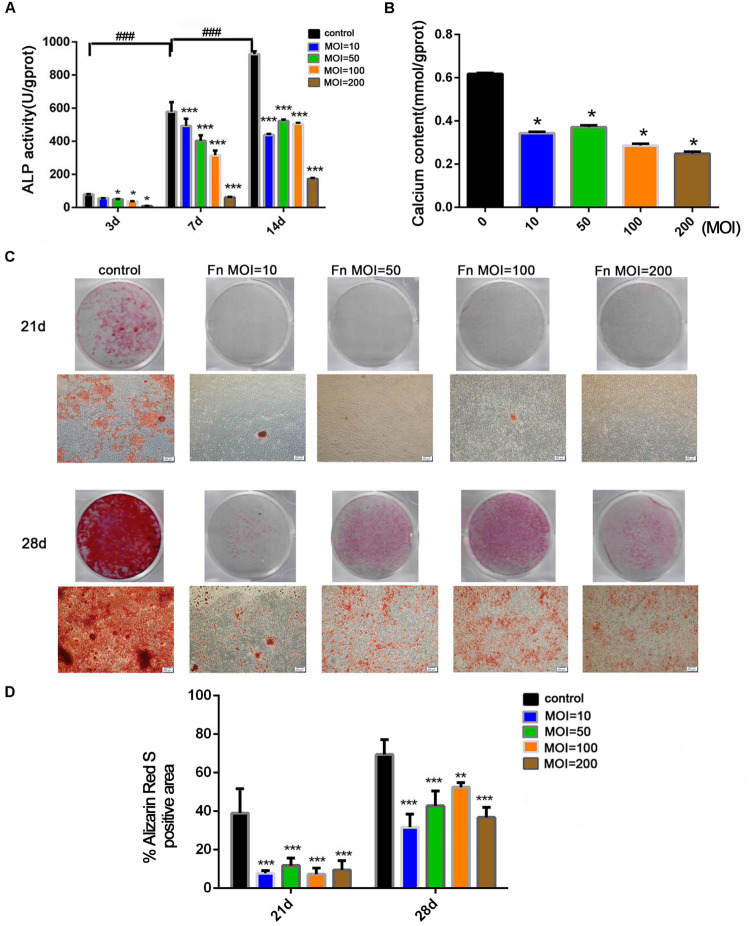
Effects of *F. nucleatum* on the osteogenesis differentiation and mineralization of osteoblasts. **(A)** ALP activity assay in osteoblasts cocultured with *F. nucleatum* (MOIs of 0, 10, 50, 100, and 200) at 3, 7, and 14 days (*n* = 3). **(B)** Calcium content assay in osteoblasts cocultured with *F. nucleatum* (MOIs of 0, 10, 50, 100, and 200) at day 21 (*n* = 3). **(C)** Representative images of alizarin red staining in osteoblasts cocultured with *F. nucleatum* (MOIs of 0, 10, 50, 100, and 200) at days 21 and 28 by digital camera or microscope (Scale bar: 200 μm). **(D)** Quantification of the percent of the area that was positive for alizarin red staining (*n* = 6). The histograms represent the mean ± SD (*p < 0.05; **p < 0.01; ***p < 0.001; ^###^*p* < 0.001).

To explore the effect of *F. nucleatum* stimulation on osteogenic differentiation and mineralization, an ALP activity assay, calcium content assay, and alizarin red staining were carried out in osteoblasts cocultured with *F. nucleatum* over the long term. ALP activity in the control group gradually increased with time from day 3 to 14, but ALP activity was dose-dependently inhibited by *F. nucleatum* in all *F. nucleatum*-stimulated groups (Figure 2A). Next, we evaluated the mineralization of osteoblasts under the effects of *F. nucleatum* by determination of the calcium content and alizarin red staining. Results of the calcium content assay on day 21 showed that *F. nucleatum* significantly inhibited intracellular calcium deposition (Figure 2B). Alizarin red staining on days 21 and 28 showed that both *F. nucleatum*-infected and uninfected osteoblasts gradually deposited additional minerals over time; a large amount of mineralized nodules was detected in the control group, while fewer nodules and minerals were detected in the infected groups (Figures 2C,D).

### *F. nucleatum* Inhibited the Expression of Osteoblast Differentiation-Related Genes and Proteins

**FIGURE 3:**
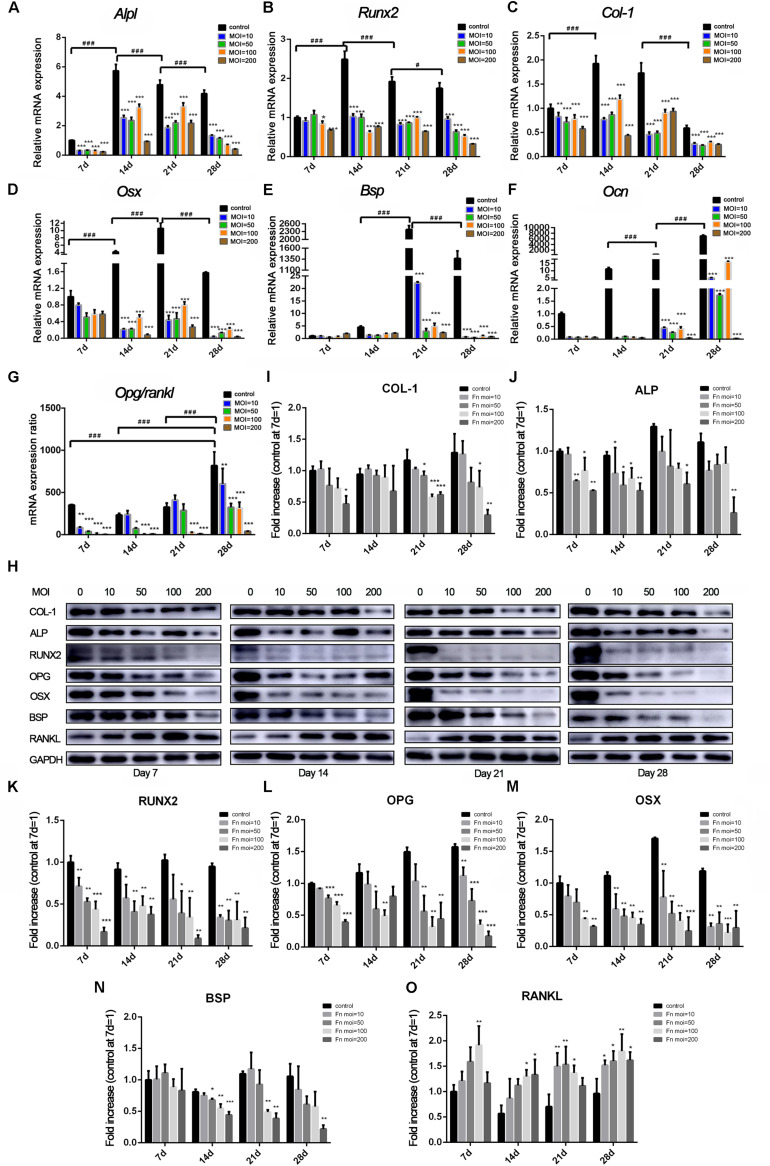
Effects of *F. nucleatum* on the expression of osteoblast differentiation-related genes and proteins. The gene expression of **(A)** *Alpl*, **(B)** *Col-1*, **(C)** *Runx2*, **(D)** *Osterix*, **(E)** *Bsp*, **(F)** *Ocn*, and (**G**) *Opg/Rankl* with *F. nucleatum* stimulation (MOIs of 0, 10, 50, 100, and 200) at 7, 14, 21, and 28 days by qRT-PCR (control at 7 day = 1). **(H)** The expression of osteogenic associated proteins ALP, COL-1, RUNX2, BSP, OSTERIX, OPG, and RANKL at days 7, 14, 21, and 28 by Western blots. The relative protein levels of COL-1/GAPDH **(I)**, ALP/GAPDH **(J)**, RUNX2/GAPDH **(K)**, OPG/GAPDH **(L)**, OSX/GAPDH **(M)**, BSP/GAPDH **(N)**, RANKL/GAPDH **(O)**. The histograms represent the mean ± SD (*n* = 3, control at 7 day = 1) (*p < 0.05; **p < 0.01; ***p < 0.001; ^#^*p* < 0.05; ^###^*p* < 0.001).

Changes in osteoblast differentiation-related transcription factors and osteogenic marker genes were measured at both the RNA (Figures 3A–G) and protein (Figures 3H–O) levels. The expression of *Alpl* (Figure 3A), *Runx2* (Figure 3B), and *Col-1* (Figure 3C) in the control group increased from day 7 to day 14, peaked at day 14, and then decreased from day 21. However, in the *F. nucleatum*-stimulated groups, expression of most of these genes was dose-dependently reduced from 14 to 28 days and some was less than one-half that of the control group. The expression of *Osx* (Figure 3D) in the *F. nucleatum*-stimulated groups was slightly decreased compared with the control group on day 7. However, its expression in the control group increased significantly and peaked on day 21, while in the experimental groups, *Osx* expression gradually decreased with time, and the fold changes in expression compared with the control group increased in a time-dependent manner. The late osteogenesis marker genes *Bsp* (Figure 3E) and *Ocn* (Figure 3F) were significantly suppressed in the *F. nucleatum*-infected groups, and the significant inhibitory effect of *F. nucleatum* gradually increased with time. The ratio of *Opg*/*Rankl* (Figure 3G) was also inhibited by *F. nucleatum*, except in the groups infected with *F. nucleatum* at a low concentration (MOI of 10) at day 14 and groups infected with *F. nucleatum* at MOIs of 10 and 50 at day 21. A heatmap of the RNA-seq data of these above genes are shown in Supplementary Figure S2, which showed a consistent changing with the qRT-PCR results. Levels of the proteins corresponding to these genes showed a similar tendency to change (Figure 3H). The levels of COL-1 (Figure 3I), ALP (Figure 3J), and BSP (Figure 3N) in groups infected with *F. nucleatum* at a high concentration (MOIs = 100, 200) were obviously decreased. In addition, the protein levels of RUNX2 (Figure 3K), OPG (Figure 3L), and OSX (Figure 3M) in almost all of the experimental groups were obviously decreased at days 7–28. In contrast, the expression of RANKL was increased due to *F. nucleatum* stimulation in the experimental groups (Figure 3O).

### Time-Dependent Whole-Transcriptome Analysis of *F. nucleatum*-Infected Osteoblasts

To analyze differences in the whole transcriptome of osteoblasts after long-term stimulation with *F. nucleatum*, we analyzed the whole transcriptomes of osteoblasts stimulated with or without *F. nucleatum* (MOIs = 0, 50) for 1, 3, 7, 14, 21, and 28 days (Supplementary Table S3). The transcriptomic output data processing steps are introduced in the materials and methods, and annotation information is listed in Supplementary Tables S4, S5.

**FIGURE 4:**
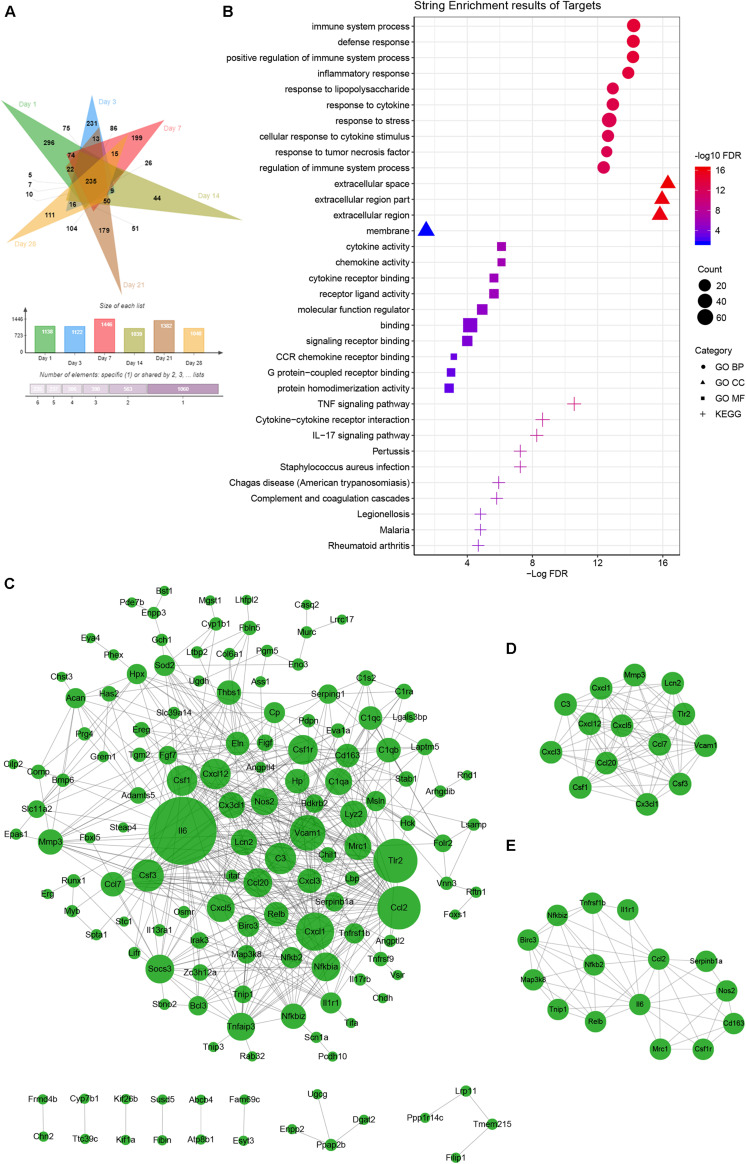
Common gene analysis in whole transcriptome of *F. nucleatum*-infected osteoblasts at days 1, 3, 7, 14, 21, and 28. **(A)** Venn diagram summarizing the overlapping analysis of 235 common DEGs among the six time points. **(B)** Functional enrichment analysis of 235 common DEGs in STRING (The different symbols represent the different enrichment patterns based on the GO and KEGG analyses). **(C)** PPI network analysis of 235 common DEGs. The size of the dot represents the degree of interactions in the network. **(D,E)** Key subnetwork analysis on the PPI network with Cytoscape’s MCODE plug-in [**(D)** MCODE score = 11.692 and **(E)** MCODE score = 7.143].

Principal component analysis of the total mRNA expression levels showed that transcriptomes of the control groups and *F. nucleatum*-infected groups were clearly separated (Supplementary Figure S3). Correlation analysis of 36 samples by the Pearson correlation coefficient (Supplementary Figure S4) showed a decreased correlation between the normal groups and *F. nucleatum*-infected groups with increasing stimulation time. To characterize the DEGs due to *F. nucleatum* infection at each time point, gene expression profiles of the control group (MOI = 0) and an experimental group (MOI = 50) at 1, 3, 7, 14, 21, and 28 days were compared, which identified 1138, 1122, 1446, 1039, 1382, and 1048 DEGs at each time point, respectively. In addition, 235 overlapping DEGs across the six time points were identified by a Venn analysis (Figure 4A). According to the enrichment analysis in the String database, these genes were annotated mainly to 396 biological process GO terms, 28 molecular function GO terms, 4 cellular component GO terms, and 35 KEGG signaling pathways (Supplementary Table S6). The top 10 enriched GO terms and KEGG pathways are shown in Figure 4B. The results suggested many inflammation- and immune-related and cytokine-related pathways were enriched during the whole *F. nucleatum* infection in osteoblasts, such as immune system process, inflammatory response, cytokine activity, TNF signaling pathway, IL-17 signaling pathway, and cytokine–cytokine receptor interaction. In addition, some carcinogenesis-related pathways, including pathways in cancer, transcriptional misregulation in cancer, Jak-STAT signaling pathway, and NF-kappa B signaling pathway, were also activated.

PPI analysis of the common DEGs was performed in a network with two key subnetworks (Figures 4C–E). The genes, *Il-6*, *Vcam1*, *Tlr2*, *Csf1r*, *Cxcl1*, *C3*, *Thbs1*, and *Socs3*, interacted more closely with other genes and played key roles in the network (Figure 4C). Most of the genes in the subnetworks (Figures 4D,E) were inflammatory cytokines and participated in TNF signaling pathways and pathways in cancer, such as *Ccl2*, *Ccl20*, *Csf1*, *Cx3cl1*, *Cxcl1*, *Cxcl3*, *Il6*, *Birc3*, *Map3k8*, *Nos2*, *Nfkb2*, *Tnfrsf1b*, and *Vcam1*.

### Whole-Transcriptome Analysis to Determine Biological Processes Enriched in Altered Genes

To further clarify the mechanisms of *F. nucleatum*-induced alterations in biological processes in osteoblasts by transcriptome analysis, the enrichment of biological process-related annotation GO terms in the DEGs was analyzed. The expression of a total of 2791 transcripts was affected during the *F. nucleatum* infection over time and enriched mainly in the biological processes of the regulation of cell adhesion, the inflammatory response, ossification, extracellular structure organization, and the negative regulation of cell proliferation pathways by Metascape analysis (Supplementary Figure S5).

**FIGURE 5:**
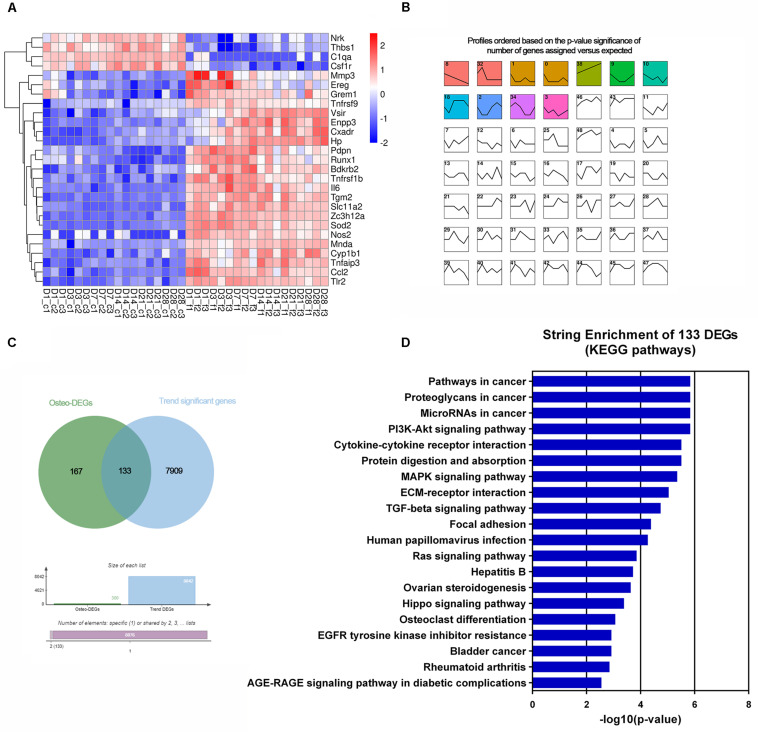
Transcriptomic analysis of the altered DEGs related to cell proliferation and apoptosis and osteogenesis. **(A)** Heatmap of 27 common cell proliferation- and apoptosis-related DEGs among six time points. **(B)** Short time-series expression miner (STEM) analysis of the DEGs in osteoblasts with *F. nucleatum* infection (MOI = 50). The colored boxes represent significantly enriched profiles (*p*-value < 0.001). **(C)** Venn analysis of osteogenisis-related DEGs and those genes whose expression showed a dynamic significant change in the long-term *F. nucleatum* infection. **(D)** Enriched KEGG pathways of the 133 dynamic significant osteogenesis-related DEGs analyzed by STRING database.

Of the cell proliferation- and apoptosis-related genes, annotation of 299 DEGs indicated negative regulation of the cell proliferation pathway (GO:0008285) and positive regulation of the cell death pathway (GO:0010942) (Supplementary Table S7). Twenty-seven genes were time-serial differentially expressed between control and *F. nucleatum*-infected osteoblasts throughout the whole process (Figure 5A). Among these genes, the expression levels of *Pdpn*, *Mnda*, *Runx1*, *Ereg*, *Enpp3*, *Grem1*, and *Vsir* were significantly upregulated, and those of *Thbs1*, *Csf1r*, *C1qa*, and *Nrk* were obviously downregulated.

**FIGURE 6:**
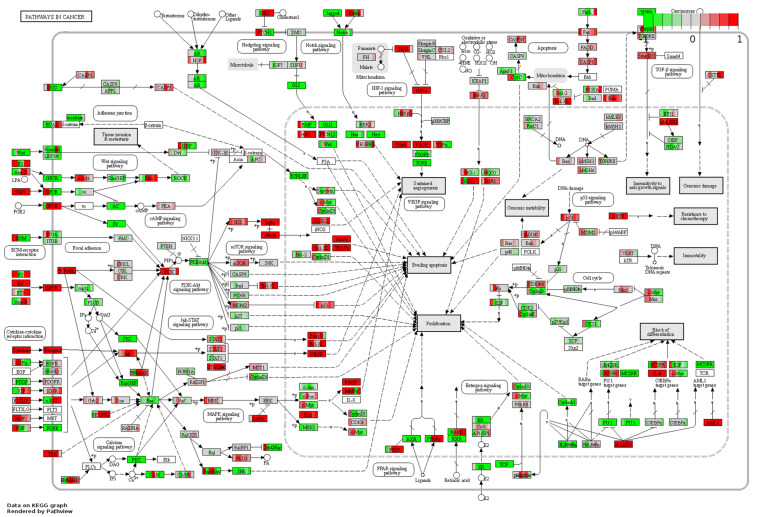
Pathview analysis of the pathways in cancer signaling pathway. Every box is divided into six parts on average, and each part represents the relative gene expression level of *F. nucleatum*-infected osteoblasts at 1, 3, 7, 14, 21, and 28 days from left to right. The red color indicates that the expression levels are upregulated after *F. nucleatum* stimulation. The green color indicates that the levels are downregulated after *F. nucleatum* stimulation. The white color indicates that the gene expression levels are not influenced by *F. nucleatum* stimulation. The gene expression levels are calculated by the log fold change in the *F. nucleatum* infected group (MOI = 50) relative to the control group at each time point.

Of the osteogenic differentiation-related genes, 120, 130, 166, 114, 146, and 149 DEGs at days 1, 3, 7, 14, 21, and 28, respectively, were shown to be involved in osteogenic processes in osteoblasts (GO:0001501, GO:0001503, and GO:0043062). A total of 300 osteogenesis-related DEGs were influenced during the whole process. To further investigate their dynamic gene expression patterns, STEM analysis of the DEGs in osteoblasts infected with *F. nucleatum* were carried out (Figure 5B). A total of 133 of these osteogenesis-related DEGs showed a dynamic and significant change in expression (Figure 5C). KEGG functional analysis revealed that these genes were not only enriched in the TGF-beta signaling pathway (an important osteogenesis pathway) but also enriched in multiple cancer-related pathways, such as pathways in cancer, microRNAs in cancer, proteoglycans in cancer, and Ras signaling pathway (Figure 5D and Supplementary Table S8). We predicted that long-term *F. nucleatum* stimulation induces alterations in intracellular gene expression and plays a role in regulating cell carcinogenesis during inhibition of the osteogenic differentiation process. In the results from the pathview analysis of pathways in cancer, the expression of a series of genes was disturbed by stimulation with *F. nucleatum*; for instance, the levels of SDF1, Jak, HGF, IL-6, AML-ETO, HIF-α, and MITF were upregulated, while those of Ras, FGFR, MSK1, RXR, PU.1, TGFβ, and GLI1 were downregulated, and the expression of many other genes was significantly changed during the infection progress (Figure 6). These changes regulated a series of genes and pathways that participate in cell survival, apoptosis, metabolism, and differentiation, which might increase the risk of cell carcinogenesis or transformation.

**FIGURE 7:**
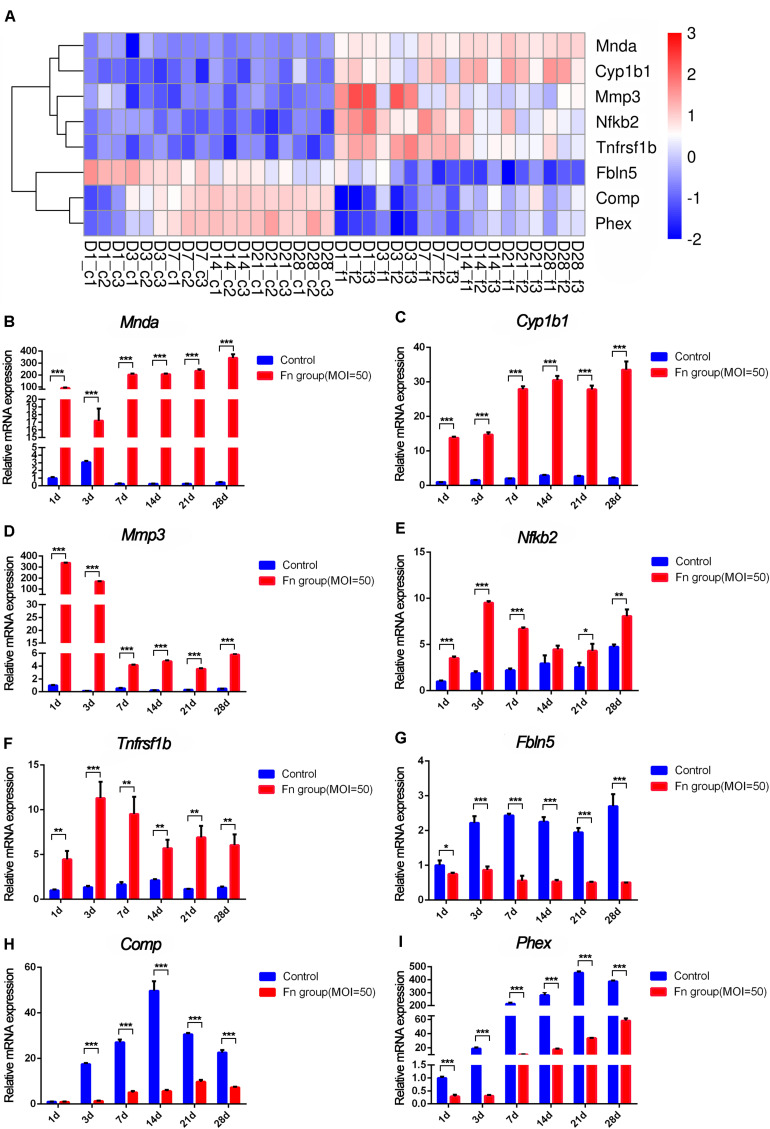
RNA-seq analysis and qRT-PCR results of the screened core osteogenic related DEGs. **(A)** Heatmap of the core osteogenic related DEGs. qRT-PCR results of *Mnda* **(B)**, *Cyp1b1* **(C)**, *Mmp3* **(D)**, *Nfkb2* **(E)**, *Tnfrsf1b* **(F)**, *Fbln5* **(G)**, *Comp* **(H)**, *Phex* **(I)** in control groups and in *F. nucleatum*-infected groups at days 1, 3, 7, 14, 21, and 28 (control at 1 day = 1) (*p < 0.05; **p < 0.01; ***p < 0.001).

Then, we screened the core genes that were both differentially expressed at all six time points and showed a continuously significant increase or decrease in expression (profile 8 and profile 38) by STEM analysis and displayed the results in a heatmap (Figure 7A). The expression levels of *Cyp1b1* and *Mnda* were upregulated, *Comp* and *Phex* were downregulated from day 1 to day 28 of stimulation with *F. nucleatum*, and their expression showed an increasing trend with time. The levels of *Mmp3*, *Nfkb2*, and *Tnfrsf1b* were upregulated, while those of *Fbln5* were downregulated in the *F. nucleatum*-infected group, and their expression showed a decreasing trend with time. The relative gene expression levels of these core dynamic DEGs (*Mnda*, *Cyp1b1*, *Comp*, *Phex*, *Mmp3*, *Tnfrsf1b*, *Fbln5*, and *Nfkb2*) were verified by qRT-PCR, and the results were identical with the significance derived from the RNA-seq data (Figures 7B–I).

## Discussion

Normal bone remodeling depends on the absorption of bone matrix by the osteoclasts and the formation of bone matrix by the osteoblasts. One of the main functions of osteoblasts is to produce the bone matrix and catalyze its calcification (Martin and Ng, 1994). The pathological environment caused by chronic inflammation often leads to deregulated bone remodeling (Zhao, 2017). Alveolar bone resorption is a main manifestation of periodontitis. In a periodontitis mouse model, *P. gingivalis* is revealed to invade alveolar osteoblasts and cause alveolar bone loss (Zhang et al., 2014). *F. nucleatum* has the strong ability to invade tissues and has been detected in oral, gastrointestinal, rheumatologic, and vascular pathologies and even the amniotic fluid of premature infants, the strain in which was proven to be the same strain found in the oral cavity (Gauthier et al., 2011; Haar et al., 2018). The obvious pathological feature of periodontitis is alveolar bone resorption, yet the specific mechanism has not been fully defined. We speculate that this effect may be related to the direct interaction between *F. nucleatum* and osteoblasts in alveolar bone, once the periodontal connective tissue is destroyed, the bacteria can directly invade the bone tissue. This study firstly evaluated the biological and molecular responses of rat osteoblasts under long-term repeated stimulation with *F. nucleatum*. Our results are the first systematic evidence that *F. nucleatum* has a direct significant inhibitory effect on osteoblasts, which provides new inspiration for understanding bone loss in periodontitis.

This study showed that *F. nucleatum* significantly inhibits the proliferation of osteoblasts by promoting caspase-8 expression and inflammatory cytokine (TNF-α, IL-6, and *Rankl*) secretion. Furthermore, upon *F. nucleatum* infection, the expression levels of a series of genes related to the negative regulation of cell proliferation and the inflammatory response were altered, and multiple inflammatory signaling pathways, including the NF-kappaB and TNF signaling pathways, were activated, as shown in our RNA-seq data. These results are consistent with other research observations, such as the findings that *Fusobacterium* stimulates Il-8 secretion and inhibits oral epithelial cell proliferation (Zhang and Rudney, 2011) and that the cell proliferation of gingival fibroblasts is inhibited via the NF-kappaB pathway (Kang et al., 2019). However, some studies have found that *F. nucleatum* can lead to DNA damage and promote oral cancer cell proliferation by the ku70/p53 pathway (Geng et al., 2020); *F. nucleatum* can also activate Toll-like receptor 4 signaling to Nuclear Factor-κB and increase the proliferation of colorectal cancer cells (Yang et al., 2017). These findings indicate that the molecular mechanisms by which cell proliferation is regulated in normal cells and in cancer cells stimulated with *F. nucleatum* are different; these specific mechanisms will be part of our follow-up research.

Heat-killed *Enterococcus faecalis* can inhibit osteoblast mineralization and decrease the expression of Runx2, osterix, Ocn, and Col-1 (Park et al., 2015). *P. gingivalis* can dose-dependently invade osteoblasts and inhibit their differentiation and mineralization by inhibiting the expression of Cbfa-1 and osterix (Zhang et al., 2010). In our study, direct *F. nucleatum* stimulation also significantly weakened osteogenic mineralization, inhibited a series of osteogenic genes and proteins, and increased Rankl expression. Sp7 is an osteoblast-specific transcription factor that is necessary for the differentiation of preosteoblasts into functional osteoblasts. It can negatively regulate the expression of Sox9 and Sox5 and prevent the differentiation of osteoprogenitor cells into chondrocytes (Zhao et al., 2008; Ishii et al., 2015). BMP is the initial inducer of osteoblast formation during bone development, and Smads are further translocated into the nucleus, where they act as transcription enhancers, interacting with Runx2 and Osterix (Nohe et al., 2004; Liu et al., 2007), thus affecting osteogenic gene transcription. Bone precursor cell types further differentiate and initiate the expression of osteoblast-specific factors, such as Alpl, Col-1, and Ocn, and promote bone formation (Rutkovskiy et al., 2016). The results of our cytology experiments and transcriptome analysis confirmed that the levels of all of these genes were significantly reduced in *F. nucleatum*-stimulated osteoblasts, leading to a decrease in osteogenesis.

Variation in gene expression patterns during infection can provide a comprehensive understanding of gene regulation and reveal the molecular mechanisms of cellular responses to bacterial invasion. We found that repeated long-term stimulation by *F. nucleatum* changed many cellular metabolic pathways and activated many cancer-related pathways in the differentiation and osteogenesis of osteoblasts, which has rarely been reported in bacteria-infected cells. Osteosarcoma is a malignant tumor that originates from normal osteoblasts or osteoblast precursors in the bone (Lillo Osuna et al., 2019). Chronic bone inflammation is one of the causes of osteosarcoma. Chronic infection and the interaction of cell surface molecules on these bacteria with the inflammatory and immune systems are important carcinogenesis mechanisms of *F. nucleatum* (Gholizadeh et al., 2017). *F. nucleatum* was reported to trigger oncogene expression in the process of gingiva-derived mesenchymal stem cell differentiation (Kang et al., 2020). CYP1B1 is an enzyme associated with angiogenesis that was found to be overexpressed in a wide variety of tumors (D’Uva et al., 2018). MNDA, which is expressed in subgroups of chronic lymphocytic leukemia, diffuse large B-cell lymphoma, and mantle-cell lymphoma and especially expressed by lymphomas derived from the marginal zone, can be used as a useful marker for nodal marginal zone lymphoma (Kanellis et al., 2009). COMP was reported to promote fibrosis in multiple organs, such as the skin, lung, and liver, and also contributes to the development of tumors, including colon cancer, breast cancer, and hepatocellular carcinoma (Li et al., 2018). A decrease in the PHEX activity leads to misregulation of osteopontin, which contributes to bone mineralization and is associated with metastasis in tumor biology (Neves et al., 2016). MMP3 can be regulated by NFAT1 and promote melanoma tumor growth and lung metastasis (Shoshan et al., 2016). These genes, which have been reported to participate in the occurrence, development, and metastasis of tumors, were also the core genes in osteoblasts whose expression changed significantly under stimulation with *F. nucleatum*, as shown by RNA-seq analysis. NFKB2 has been observed to be upregulated in gastric cancer tissues (Rossi et al., 2019), and its altered expression can collaborate with heterozygous Kras^MUT^ to drive tumorigenesis but decreases the metastatic potential of pancreatic ductal adenocarcinoma (Mueller et al., 2018). The upregulation of Tnfrsf1b, which belongs to the TNF receptor superfamily, has also been found in gastric cancer tissues (Rossi et al., 2019), and Tnfrsf1b can promote colorectal carcinogenesis (Liu et al., 2018). Fbln5 can inhibit epithelial cell proliferation and carries out tumor-promoting and tumor-protective functions in breast lesions (Karanis et al., 2019). We speculate that long-term direct stimulation with *F. nucleatum* and the inflammatory environment lead to changes in the intracellular environment and intracellular signaling pathways, which may play a role in the development of osteosarcoma; this might be another reason for the reduced osteogenic ability of osteoblasts. Further *in vivo* and clinical data to validate the effect of *F. nucleatum* on carcinogenesis will be obtained in our future studies.

## Conclusion

In summary, the pathogenic effect of *F. nucleatum* on osteoblasts is a complex and long-term process involving multiple genes and pathways related to cell survival and differentiation. We confirmed that *F. nucleatum* can dose- and time-dependently inhibit cell proliferation, promote cell apoptosis and inflammatory cytokine secretion, and inhibit osteogenic differentiation and mineralization. Inflammation-related genes and pathways are continuously activated during the chronic infection. Long-term *F. nucleatum* stimulation may increase the risk of cell carcinogenesis by altering many inflammation- and cancer-related genes and pathways, such as *Mnda*, *Cyp1b1*, *Comp*, *Phex*, *Mmp3*, *Tnfrsf1b*, *Fbln5*, and *Nfkb2*.

## Data Availability Statement

The datasets presented in this study can be found in online repositories. The names of the repository/repositories and accession number(s) can be found in the article/ Supplementary Material. If any other data are needed, please contact the corresponding authors.

## Ethics Statement

The animal study was reviewed and approved by Medical Ethical Committee of Stomatology School, Shandong University (Protocol Number: 20180102).

## Author Contributions

QF and JZ conceived, designed, and supervised this study. HG, FY, and MY collected the samples. HG, DT, WK, and JY performed the experiments. HG and TS analyzed and interpreted the data. HG and QF wrote the manuscript. All authors commented on the manuscript.

## Conflict of Interest

The authors declare that the research was conducted in the absence of any commercial or financial relationships that could be construed as a potential conflict of interest.

## Footnotes

1 http://genome.ucsc.edu/

## Supplementary Material

FIGURE S1 Representative cell populations images in flow cytometry analysis of cell apoptosis in osteoblasts with or without *F. nucleatum* stimulation (MOI = 0, 10, 50, 100, and 200) at 2, 6, 12, and 24 h, respectively; stained with Annexin V-FITC/PI. The proportion of non-apoptotic cells (Lower left quadrant: Annexin V-FITC^–^/PI^–^), early apoptotic cells (Lower right quadrant: Annexin V-FITC^+^/PI^–^), late apoptotic/necrotic cells (Upper right quadrant: Annexin V-FITC^+^/PI^+^) and dead cells (Upper left quadrant: Annexin V-FITC^–^/PI^+^). The black color indicates the proportion of normal cells, and the red color indicates the proportion of total apoptotic cells.

FIGURE S2 Heatmap of the osteoblast differentiation-related genes by RNA-seq data.

FIGURE S3 PCA analysis of 36 samples in RNA-seq analysis.

FIGURE S4 Pearson correlation coefficient between 36 samples.

FIGURE S5 Bar graph of the top 100 functionally enriched biological process GO terms of the total 2791 DEGs in the six time points by Metascape analysis.

TABLE S1 Primer sequences for quantitative real-time PCR (qRT-PCR).

TABLE S2 Primary antibodies information for western blotting.

TABLE S3 Sample collection and groups information.

TABLE S4 Reads statistics of sequencing data and reference genome comparison.

TABLE S5 Statistics of species annotation information.

TABLE S6 Functional enrichment results of 235 common DEGs among six time points by STRING database analysis (Go terms and KEGG pathways).

TABLE S7 Results of enriched biological process GO terms of the total 2791 DEGs in all six time points by Metascape analysis (Top 20 pathways).

TABLE S8 KEGG enrichment analysis results of 133 dynamic significant osteogenic-related DEGs.
