# Supplementary figures and images for: The Pathogenic Effects of Fusobacterium nucleatum on the Proliferation, Osteogenic Differentiation, and Transcriptome of Osteoblasts
Source: Front Cell Dev Biol. 2020 Sep 11;8:807. doi: 10.3389/fcell.2020.00807 (PMC7517582; doi:10.3389/fcell.2020.00807)

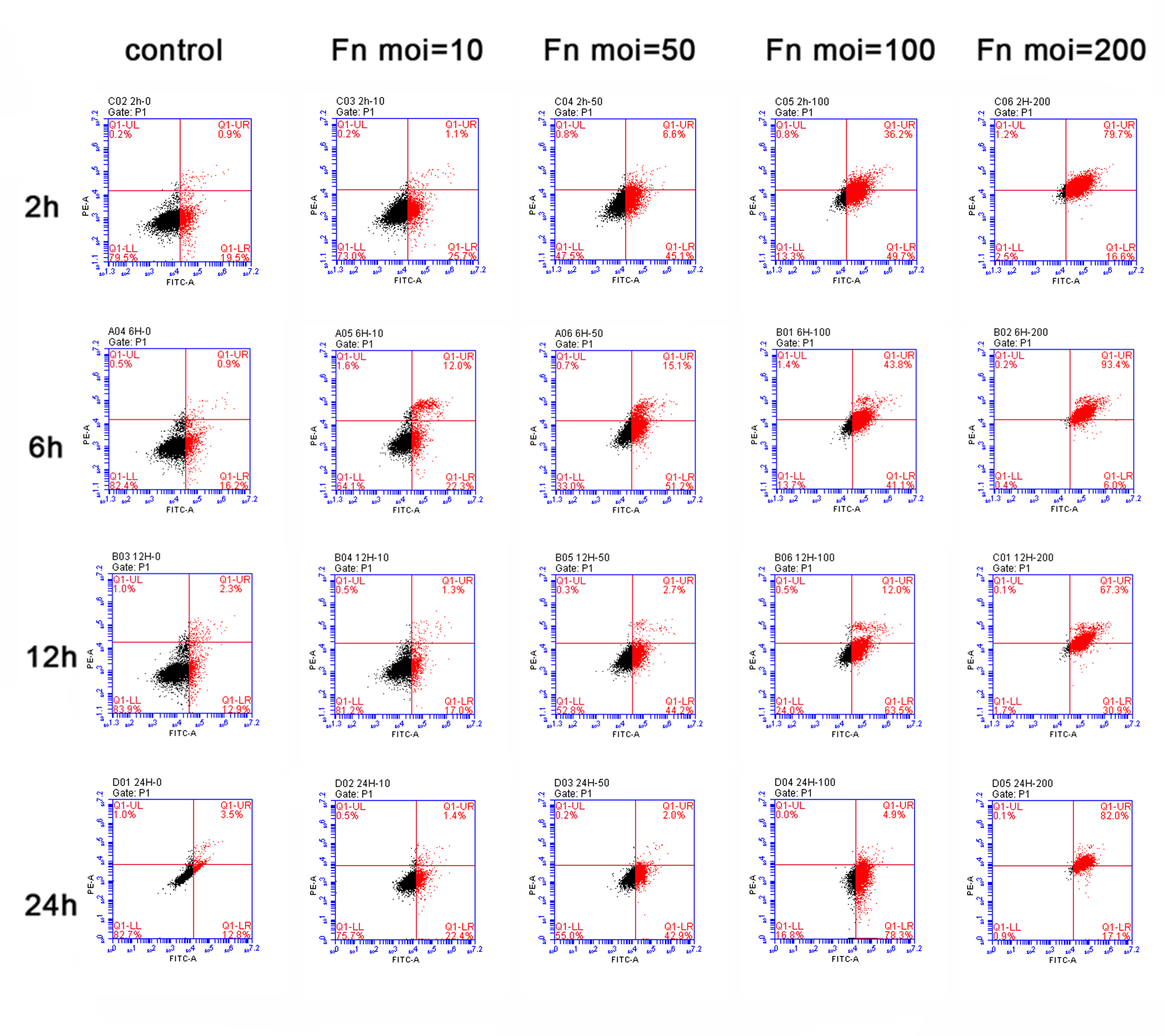

Supplement: FIGURE S1 — Representative cell populations images in flow cytometry analysis of cell apoptosis in osteoblasts with or without F. nucleatum stimulation (MOI = 0, 10, 50, 100, and 200) at 2, 6, 12, and 24 h, respectively; stained with Annexin V-FITC/PI. The proportion of non-apoptotic cells (Lower left quadrant: Annexin V-FITC–/PI–), early apoptotic cells (Lower right quadrant: Annexin V-FITC+/PI–), late apoptotic/necrotic cells (Upper right quadrant: Annexin V-FITC+/PI+) and dead cells (Upper left quadrant: Annexin V-FITC–/PI+). The black color indicates the proportion of normal cells, and the red color indicates the proportion of total apoptotic cells. [file Image_1.TIF]

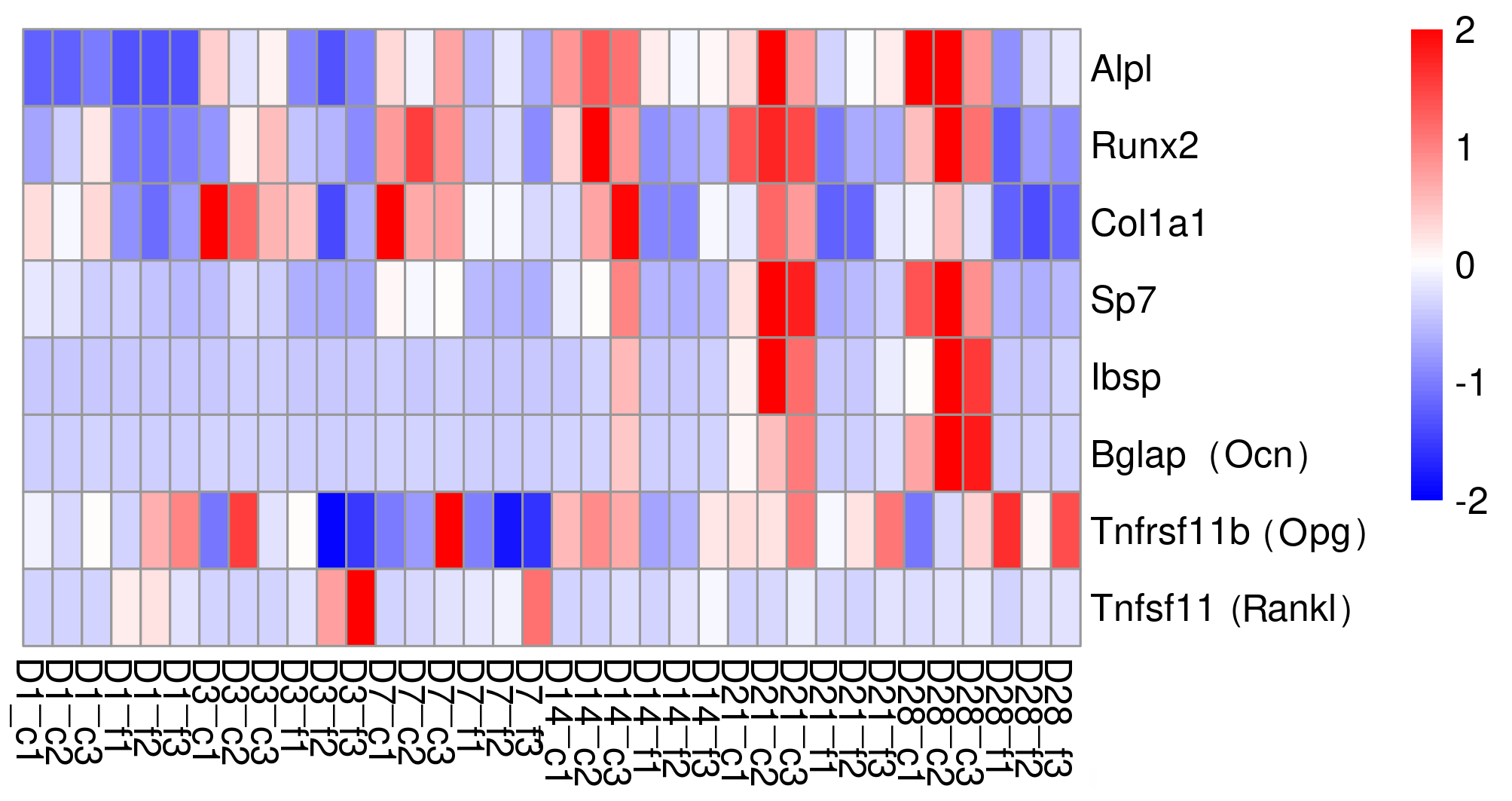

Supplement: FIGURE S2 — Heatmap of the osteoblast differentiation-related genes by RNA-seq data. [file Image_2.TIF]

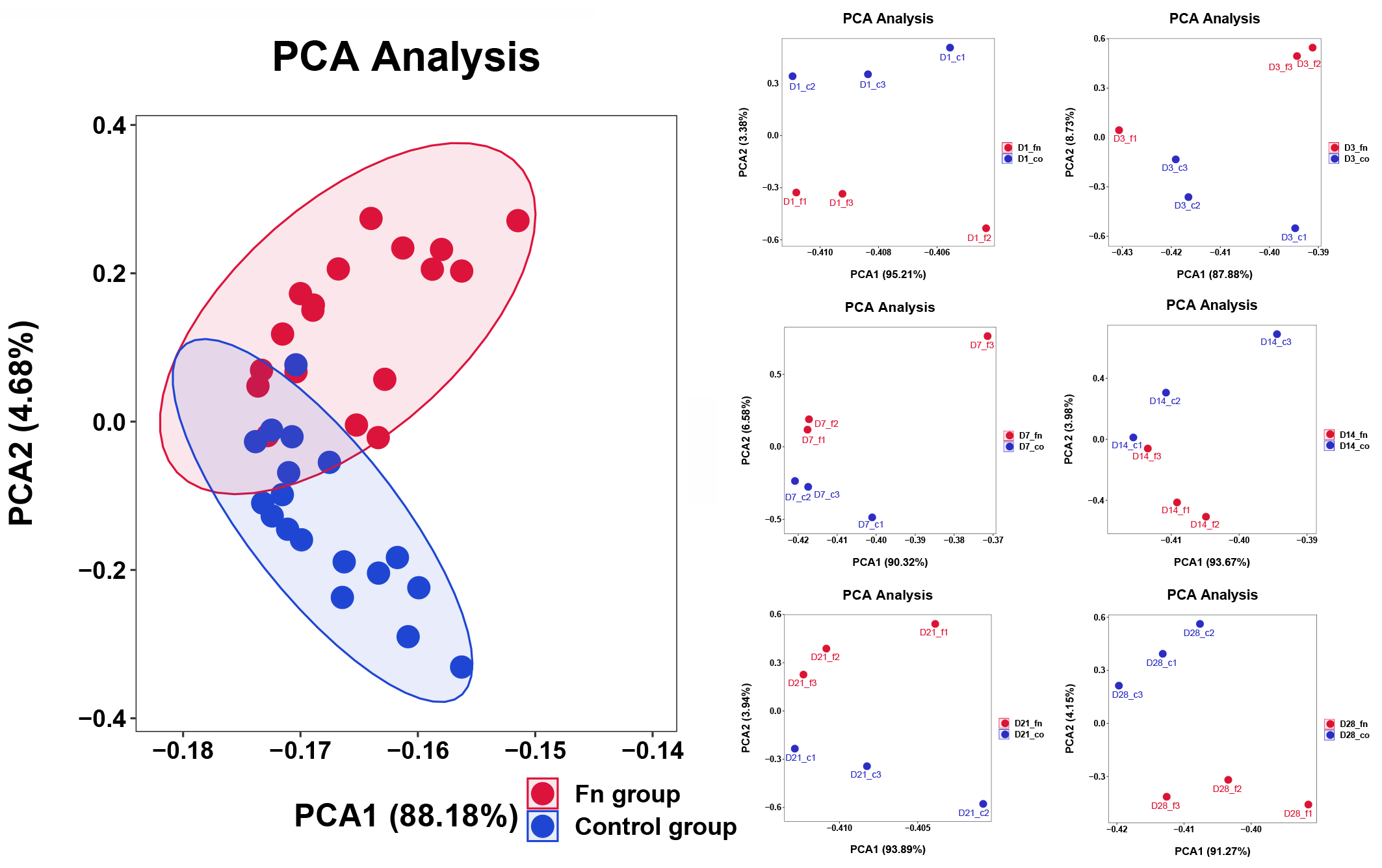

Supplement: FIGURE S3 — PCA analysis of 36 samples in RNA-seq analysis. [file Image_3.TIF]

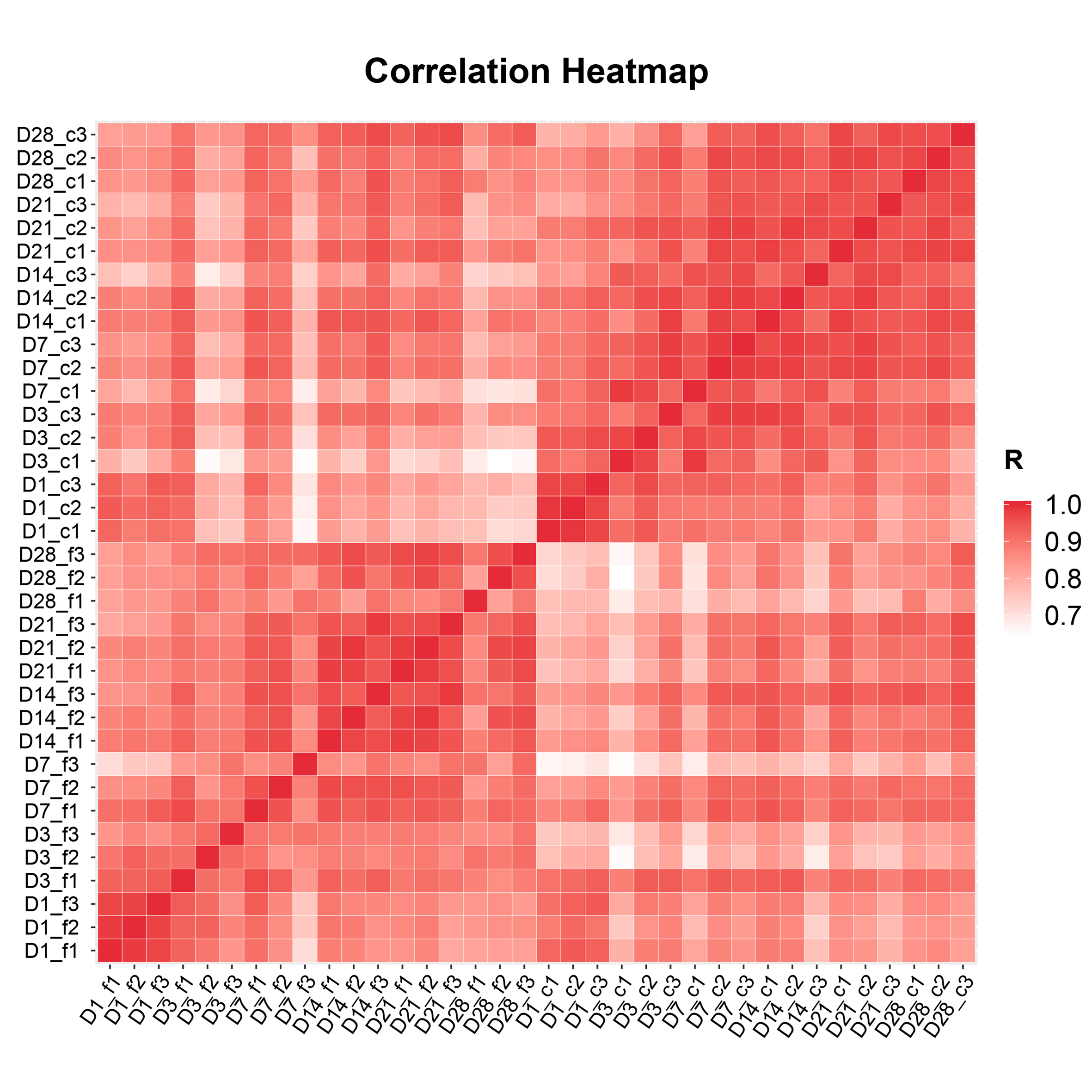

Supplement: FIGURE S4 — Pearson correlation coefficient between 36 samples. [file Image_4.TIF]

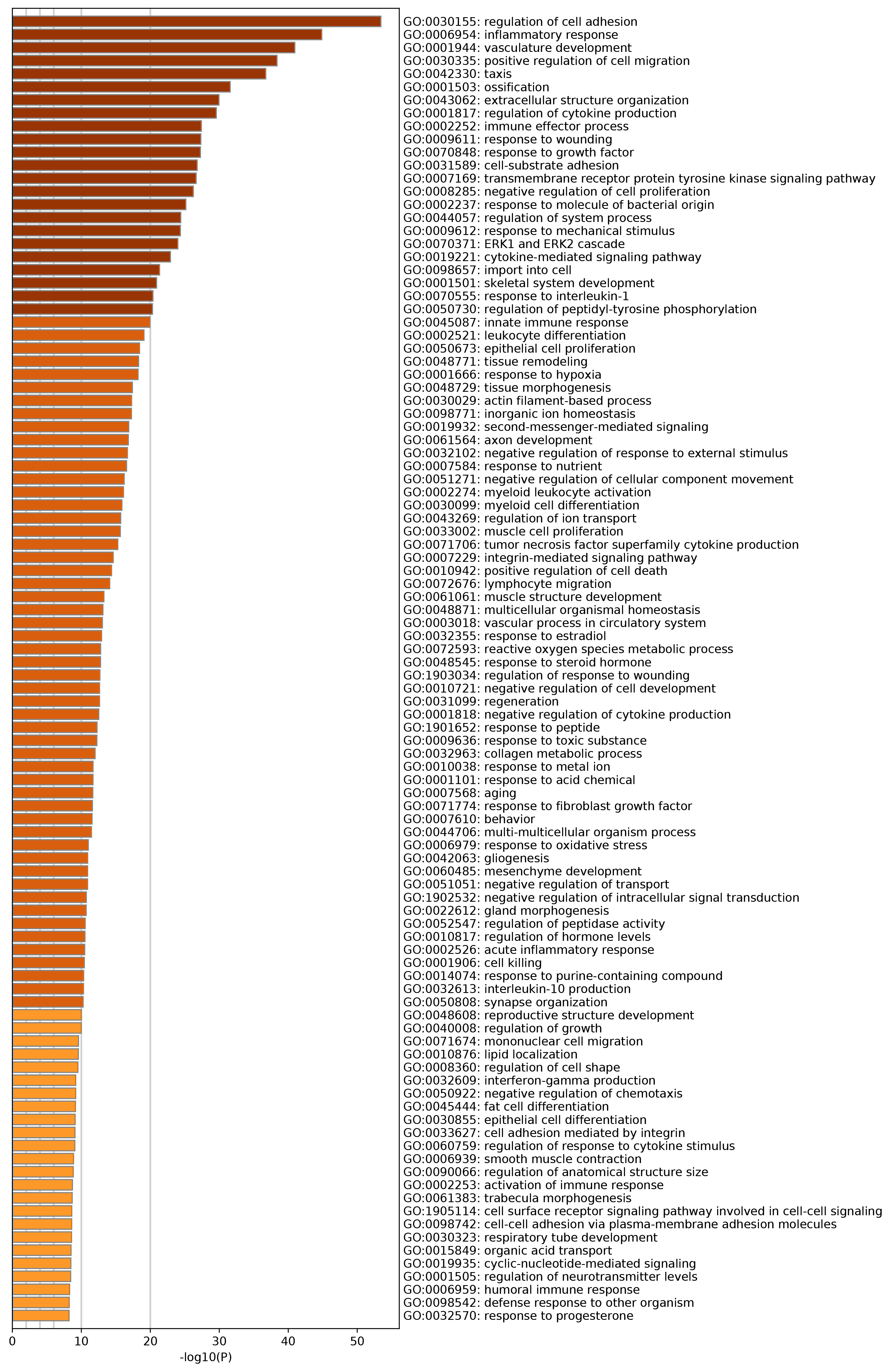

Supplement: FIGURE S5 — Bar graph of the top 100 functionally enriched biological process GO terms of the total 2791 DEGs in the six time points by Metascape analysis. [file Image_5.TIF]
