# Supplementary material for: The Pathogenic Effects of Fusobacterium nucleatum on the Proliferation, Osteogenic Differentiation, and Transcriptome of Osteoblasts
Source: Front Cell Dev Biol. 2020 Sep 11;8:807. doi: 10.3389/fcell.2020.00807 (PMC7517582; doi:10.3389/fcell.2020.00807)
Supplement: TABLE S1 — Primer sequences for quantitative real-time PCR (qRT-PCR). [file Table_1.DOC]

**Table S1. Primer sequences for quantitative real-time PCR (qRT-PCR)**

| Gene | Primer sequences | |
| --- | --- | --- |
|  | 5ˊ~3ˊForward | 5ˊ~3ˊReverse |
| Caspase-8  *Col-1*  *Osx*  *Alpl*  *Rankl*  *Opg*  *Runx2*  *Ocn*  *Bsp*  *Mnda*  *Cyp1b1*  *Comp*  *Phex*  *Mmp3*  *Tnfrsf1b*  *Fbln5*  *Nfkb2*  β-actin | GGAAGGATCGACGATTACGA  CGAGTATGGAAGCGAAGGTT  CTGGGAAAAGGAGGCACAAAGAA  AGATGGAGAAGTTCCCCTTTG  GCCGTGCAAAGGGAATTACAA  GCACGAGCCTTATCCCATTTGTAG  CCCAACTTCCTGTGCTCC  TCTGACAAAGCCTTCATGTCC  AGCTGACCAGTTATGGCACC  GGAGGCTCACCCAACAACTT  ATCTCAACCGCAACTCCAACT  CCTGCGTTCTAGTGCTCGC  TGCGCGTCCCACAATACTT  CAGGCATTGGCACAAAGGTG  TTGAACCAAGCATCACGGGT  ATGGCTATGGCTGAGATTCCG  TCTCGACCTCCACCGGATCTTT  AGCCTTCCTTCTTGGGTATG | TGCAGCAGATGAAGCAGTCT  CCACAAGCGTGCTGTAGGT  GGCAAAGTCAGACGGGTAAGTAG  ACACAAGTAGGCAGTGGCAGT  ATGGTGAGGTGAGCAAACGG  ATAAGAGGGCGCATAGTCAGTAGA  AGTGAAACTCTTGCCTCGTC  GAAGCCAATGTGGTCCGCTA  TTCCCCATACTCAACCGTGC  TGCCCAATCCAGCATCATCT  CTTTAGCACCCACTCTTGGCT  CTCTCTCACGTCTTGCAGCG  AGGCAGCAGAGTTGTTGTCC  GTGGGTCACTTTCCCTGCAT  TCGCCAGTCCTAACATCAGC  CCCACTCCCAAACCAAGACT  CAGCTTCCCAGAGTTTCAGACG  GGTCTTTACGGATGTCAACG |
