# Supplementary material for: The Pathogenic Effects of Fusobacterium nucleatum on the Proliferation, Osteogenic Differentiation, and Transcriptome of Osteoblasts
Source: Front Cell Dev Biol. 2020 Sep 11;8:807. doi: 10.3389/fcell.2020.00807 (PMC7517582; doi:10.3389/fcell.2020.00807)
Supplement: TABLE S2 — Primary antibodies information for western blotting. [file Table_2.DOC]

**Table S2. Primary antibodies information for Western blotting**

| Antibody  (Catalog number) | Company | dilution | Molecular Weight (kDa) |
| --- | --- | --- | --- |
| ALP (ab108337) | Abcam | 1:10000 | 74 |
| COL1 (WL0088) | WanLei, Shenyang, Liaoning, China | 1:1000 | 130 |
| Runx2 (#12556) | Cell Signaling Technology, Danvers, MA, USA | 1:1000 | 60 |
| Osterix (ab209484) | Abcam，Cambridge, UK | 1:1000 | 45 |
| Opg(DF6824 ) | Affinity, Cincinnati, OH, USA | 1:1000 | 46 |
| BSP (DF7738) | Affinity, Cincinnati, OH, USA | 1:1000 | 36 |
| Rankl(NHA14594) | Novogene, Tianjin, China | 1:1000 | 35 |
| GAPDH(10494-1-AP) | Proteintech, Chicago, IN, USA | 1:10000 | 36 |
